# Trees as a metaphor to understand relationships in biology

**DOI:** 10.1371/journal.pbio.3002681

**Published:** 2024-05-28

**Authors:** Roland G. Roberts, on behalf of PLOS Biology staff editors

**Affiliations:** Public Library of Science, San Francisco, California, United States of America and Cambridge, United Kingdom

## Abstract

The phylogenetic tree has been a core conceptual tool for evolutionary biology for nearly 200 years. This editorial explores the role of the tree as a metaphor, discussing two new PLOS Biology Essays that look to the future.

We all know what trees look like; a single sturdy trunk, splitting into progressively thinner branches that end in a thousand leaf-bearing twigs. We also have some idea of the process by which they arise from their single origin, via linear growth through time, ramified by a series of simple forks.

Many branching organs arise in biology through related processes, and some of these bear the name “tree” to reflect this fact, whether in English (“bronchial tree”) or other languages—“dendritic arbor” manages to incorporate both Greek and Latin words for tree.

But there are other aspects of life on Earth where the tree has taken a more metaphorical turn. With its own roots in family trees and taxonomic trees, the phylogenetic tree first appears in a now-famous 1837 notebook jotting by Charles Darwin (Fig 1A), with more literally tree-like representations by scientists such as Ernst Haeckel (Fig 1B).

**Fig 1:**
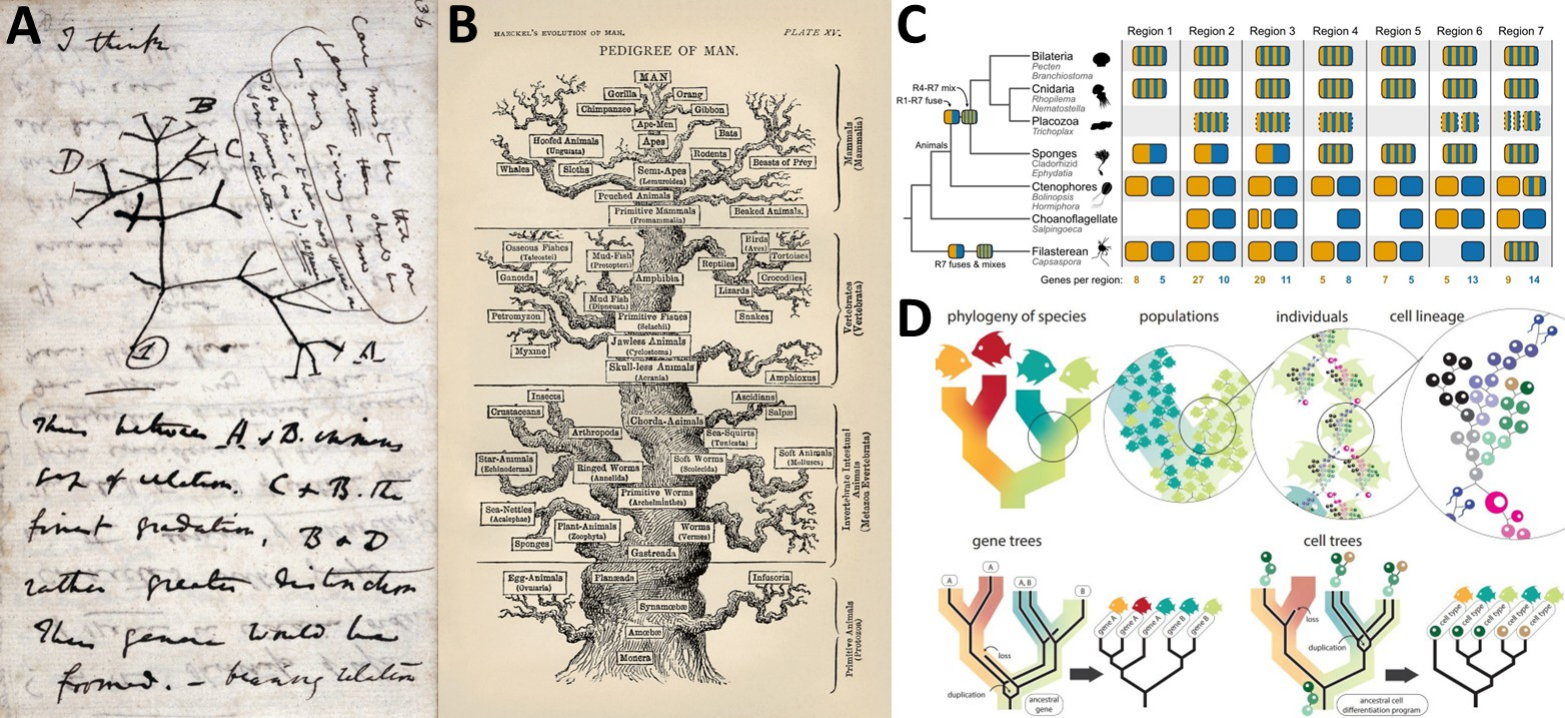
Nearly two centuries of phylogenetic trees. A. The iconic page from Charles Darwin’s 1837 notebook. B. Ernst Haeckel’s very literal and anthropocentric phylogenetic tree from his 1879 book “The evolution of man.” C. Steenwyk and King’s depiction [1] of the use of synteny arguments by Schultz et al. to probe the deep evolutionary history of animals [2]. D. Church *et al*. propose intersecting multiple trees to generate a tree of cellular life. Images are from Wikimedia Commons (A and B, public domain), pbio.3002632, pbio.3002632, respectively.

Darwin’s conceptual leap was that this tree did not represent a mere rigid taxonomy, categorising a fait accompli creation in hierarchical form, but rather that it arose by a process of genotypic and phenotypic variation over enormous tracts of time and serial division through speciation. The metaphor works at the level of the process, as well as the form.

Nearly 190 years later, our journals are full of phylogenetic trees of staggering complexity, and they remain a central tool of evolutionary biology. However, there are some aspects in which the tree metaphor has its limitations; phylogenetic trees have been heavily pruned by extinction, branch points can be knotted and reticular rather than neat and binary, and branches can fuse and exchange material. We need to ensure that the metaphor serves us without constraining us, but on the whole, it largely holds water.

The types of character data that are used to infer the relationship between the branch-tips have moved with the technology, starting with gross anatomical features and progressing via antigen cross-reactivity and gene sequences to entire genomes. As higher-quality, chromosome-level genome assemblies become more widely available, some researchers are using synteny data (roughly speaking, chromosomal gene order) to tease apart particularly tricky branches in the Tree of Life. As Steenwyk and King describe in this issue of PLOS Biology [1], the benefit of synteny data is the rarity and specificity of the changes that are studied. The assumption that the observed changes in synteny are unique reduces the chances of confusing convergence for genuine relatedness. Steenwyk and King use two particularly thorny phylogenetic problems (the base of the animal tree [2], Fig 1C, and the relationship between major groups of teleost fish [3]) as case studies of how recent consideration of synteny has allowed us to disentangle relationships that had previously been challenging to disambiguate.

By contrast, Church *et al*. consider an orthogonal tree that is present in multicellular organisms [4]—the series of binary divisions that generate the trillions of cells in our own bodies from a single zygotic cell, including the generation of distinct cell types through differentiation. They recognise that the cellular trees of different animals could be combined with species and gene phylogenetic trees to generate an overarching tree of cellular life (Fig 1D). Thus, to use a Shakesperean example, a newt’s eyeball and a frog’s toe are both derived from a single ancestral cell (the zygote of their last common ancestor) by processes of speciation and differentiation. Again, technological advance is in the driving seat, as Church *et al*. propose that this could be formalized by leveraging the copious single-cell transcriptome (scRNA-seq) datasets that are increasingly available. The use of phylogenetic methods to explore comparative scRNA-seq data promises a new level of resolution in evolutionary developmental biology.

Both of the enabling technologies (widespread availability of chromosome-level genome assemblies and scRNA-seq data) have arisen very recently, so who knows what will we be doing with trees in another 200 years…?
